# Micronutrient intake inadequacy and its associated factors among lactating women in Bahir Dar city, Northwest Ethiopia, 2021

**DOI:** 10.1371/journal.pone.0271984

**Published:** 2022-07-27

**Authors:** Mahider Awoke, Yonatan Menber, Hanna Demelash Desyibelew, Samuel Dagne, Tewodros Tadesse, Yosef Wassihun

**Affiliations:** 1 Department of Public Health, College of Health Science, Injibara University, Injibara, Ethiopia; 2 Department of Nutrition and Dietetics, School of Public Health, College of Medicine and Health Science, Bahir Dar University, Bahir Dar, Ethiopia; 3 Department of Surgery, Felege Hiwot Comprehensive Specialized Hospital, Bahir Dar, Ethiopia; 4 Department of Health Promotion and Behavioral Science, School of Public Health, College of Medicine and Health Science, Bahir Dar University, Bahir Dar, Ethiopia; Università degli Studi di Milano, ITALY

## Abstract

**Background:**

Inadequate intake of micronutrients in lactating women was prevalent worldwide. In particular, to our knowledge, there has been little report concerning Ethiopian lactating women regarding their micronutrient intake. Our objective was to assess micronutrient intake inadequacy and its associated factors among lactating women in Bahir Dar city, Northwest Ethiopia, 2021.

**Methods:**

Community-based cross-sectional study was conducted from February 15 to March 05, 2021. Four hundred thirteen respondents were selected through systematic random sampling. Data were collected by interviewer-administered semi-structured questionnaire and a single multiphasic 24 hours dietary recall was used to assess dietary assessment. Data entry and analysis were carried out using EpiData and SPSS respectively. The ESHA food processor, Ethiopian food composition table, and world food composition table have used the calculation of nutrient values of the selected micronutrient. The nutrient intakes were assessed by Nutrient Adequacy Ratio (NAR) and Mean Adequacy Ratio (MAR). Multivariable binary logistic regression analysis was done to identify the factors of overall micronutrient intake inadequacy.

**Result:**

The overall prevalence of micronutrient intake inadequacy across 12 nutrients was 39.9% [95% CI (34.9, 45.0)]. The inadequate intake of vitamin A was 98.2%. Similarly, the inadequate intake of B vitamins ranges from 13.4% to 68.5%. The insufficient intakes of calcium, iron, and zinc were 70.9%, 0%, and 4.7%, respectively. Around 36 and 91.6% of the respondents had inadequate intake of selenium and sodium, respectively. On multivariable logistic regression analysis; Being divorced was 2.7 times more likely to have overall micronutrient intake inadequacy than being married [AOR = 2.71, 95% CI (1.01, 7.33)]. The odds of overall micronutrient intake inadequacy were 2.6 higher in merchants than in housewives [AOR = 2.63, 95% CI (1.40, 4.93)]. Lactating women who had poor nutritional knowledge were 2.7 times more likely to have overall micronutrient intake inadequacy than those who had good nutritional knowledge [AOR = 2.71, 95% CI (1.47, 4.99)].

**Conclusion and recommendation:**

Overall, the micronutrient intake in lactating women was lower than the recommended levels. Therefore; educating lactating women about appropriate dietary intake is essential.

## Introduction

Nutritional adequacy is defined as the sufficient intake of essential nutrients, needed to fulfill nutritional requirements for optimal health and the requirement for a given nutrient may be at a lower or higher intake amount [1]. One of the aims of nutritional assessment both at the individual and at the population level is to evaluate to what extent food and nutrient intake are ‘adequate’. Ideally, the comparison between the requirement and the intake for every nutrient of interest for a certain individual or population should allow one to conclude whether the diet of that individual/population was adequate or not adequate [2].

The Nutrient Adequacy Ratio (NAR) is an index of adequacy, which compares the individual’s daily intake of a nutrient with the corresponding current recommended allowance for that nutrient given the respondent’s age and sex. The Mean Adequacy Ratio (MAR) calculates the average of the NAR values for the selected nutrients for certain individuals [3, 4].

Breastfeeding imposes additional nutrient demands on lactating women to cover both the energy cost of milk production and the nutrients secreted in breast milk. As a consequence, the requirements for some micronutrients are increased by 50% or more during lactation [5]. Globally the prevalence of inadequacy was highest (>40%) for calcium, vitamin A, niacin, vitamin B-6, and vitamin B-12, all micronutrients provided by dietary sources [5, 6] in addition to that approximately 50% world population of lactating women had inadequate intake of iron and/or zinc [7–9]. In sub-Saharan Africa, the prevalence of inadequacy of micronutrients ranges from 71 to 100% for vitamin A, vitamin C, vitamin B1, B2, B3, B12, folate, and calcium, and above 30% of the sub-Saharan Africa population of lactating women had inadequate intake of iron and/or zinc [10, 11]. Different studies conducted in Nepal, Indonesia, China, Brazil, New Zealand, and Nigeria revealed that the intake of micronutrients was inadequate [5, 7–9, 12–15].

Micronutrient deficiencies can negatively impact the health of the mother, as well as the health of the newborn baby [16–18]. Inadequate maternal diet during the lactation period can lead to low micronutrient secretion in breast milk, which will often have a deleterious long-term effect on the health of the child, extreme birth defects, undeveloped cognitive capacity, decreased physical working capacity, and overall reproductive performance, maternal and infant morbidity and mortality, and childhood blindness are some of the Consequences of inadequate maternal nutrient intake [18–20].

Globally there are many launched nutrition intervention programs to eliminate all types of malnutrition by 2025 and 2030 [21, 22]. The Ethiopian government has introduced numerous intervention programs to alleviate nutrition-related health problems, with maternity continuum care (integrated components of maternal health service from pregnancy to the postpartum period to improve maternal, neonatal, and child health), micronutrient supplementation for pregnant and lactating women, the introduction of Health Extension Program (HEP) packages, national Food and Nutrition Policy; National Nutrition Program (NNPII), and nutrition education for dietary diversification, yet overall, nutrition is inadequate.

The number of micronutrient intake inadequacy studies in lactating women was scarce in Ethiopia. Thus, the question of the prevalence of micronutrients and the overall prevalence of the studied micronutrient inadequacy among lactating women were not answered. Therefore, the aim of this study was to assess micronutrient intake inadequacy and its associated factors among lactating women in Bahir Dar city, Northwest Ethiopia, 2021.

## Methods and materials

### Study setting and study design

The study was conducted in Bahir Dar city, Northwest Ethiopia, which is the capital city of the Amhara region and it is 565 km far from Addis Ababa, the capital city of Ethiopia. A community-based cross-sectional study design was conducted from February 15 to March 05, 2021.

### Population and eligibility criteria

All lactating women who were living in Bahir Dar city and lactating women who were living in the selected kebeles of Bahir Dar city were considered as source and study population respectively. Lactating women who were between the ages of 19–49 years old, after 45 days postpartum, breastfeeding their infants during the data collection period, and who had been living for six months and above in Bahir Dar city were included from the study. Lactating women who were celebrating festivals (e.g. marriage, birth dates, and Christianity) in the last 24hrs were excluded from the study.

### Sample size determination and sampling techniques

The calculated sample size was 413 after adding a 10% non-response rate and using the epi info software by considering the assumption of confidence limit (5%), and the overall prevalence of micronutrient intake inadequacy(42.2%) among lactating women in Samre Woreda, South Eastern Zone of Tigray, Ethiopia [23].

There are 26 Kebeles in Bahir Dar city. Of them, 8 kebeles were selected randomly by the lottery method. According to the Bahir Dar city administration office report of 2020, there were a total of 1059 lactating women in Bahir Dar city administration at the time of the study(Bahir Dar city administration office. 2020 annual report. unpublished) [24]. In the selected kebeles; 2022 study participants were found. Of 2022 lactating women, 413 study participants were selected by systematic random sampling method. The value of ‘K’ is calculated from N/n 2022/413 = 4; Where N = study population, n = sample size. The study participants were proportionally allocated.

### Operational definitions

**Poor knowledge:** The respondent answers less than 50% of knowledge questions(0–11) [25],

**Medium knowledge:** The respondent answers 50% to 80% of knowledge questions(12–19) [25],

**Good knowledge:** The respondent answers more than 80% of knowledge questions(≥20) [25].

**Household Food Insecurity Accesses Scale (HFIAS)**: Can be scored and classified as; food secure, and food insecure [26].

**Adequate Intake:** The micronutrient intake is equal to or greater than the RDA/RNI/Adequate intake level (AI).

**Nutrient Adequacy Ratio (NAR)**: The actual micronutrient intake per day for a particular micronutrient divided by the RDA of that micronutrient.

**Mean Adequacy Ratio (MAR)**: The summation of the Nutrient Adequacy Ratio (NAR) of all micronutrients included in the study, divided by the total number of micronutrients.

**Recommended Dietary Allowances/Reference Nutrient Intake (RNI):** this is the daily intake, which meets the nutrient requirements of almost all (97.5 percent) lactating women [27].

**Portion Size**: The amount of a food item consumed at a time.

**Kebele:** The smallest unit of administration in the government structure [28, 29].

### Data collection tools and procedures

The data were collected by six trained Public Health Officers and two BSc Nurses and supervised by two Public Health Officers. Socio-demographic and economic factors, knowledge-related factors, and health-related factors were gathered using a standardized structured questionnaire which was prepared after reading various literature, and the dietary data were assessed by the Food and Agriculture Organization of the United Nations (FAO) Standardized tool [30]. Food insecurity was measured by the Household Food Insecurity Access Scale (HFIAS) which consists of nine occurrence questions that represent a generally increasing level of severity of food insecurity (access), and nine “frequency-of-occurrence” questions that were asked as a follow-up to each occurrence question to determine how often the condition occurred during the previous 4 weeks (last one month) [26]. The wealth index of the households was assessed based on household assets. Information on the wealth index was based on data collected in the household questionnaire. Each household asset for which information was assigned a weight or factor score generated through principal components analysis. These standardized scores are then used to create the breakpoints that define five groups of wealth quintiles poorest, poor, middle, rich, and richest [31]. Knowledge of the respondent about the requirement of additional meals during lactation, the importance of iron-folic acid supplementation, nutrient intake benefits, and its food sources were assessed. Overall 10 knowledge assessing yes/no and multiple response questions with a total score of 24 were used [32].

### Dietary assessment

A single multiple-pass 24 hours recall was used in the community. Women were asked to name all foods and beverages eaten during the previous day (24 hours), including everything consumed outside the home as well as the cooking method.

**Fig 1:**
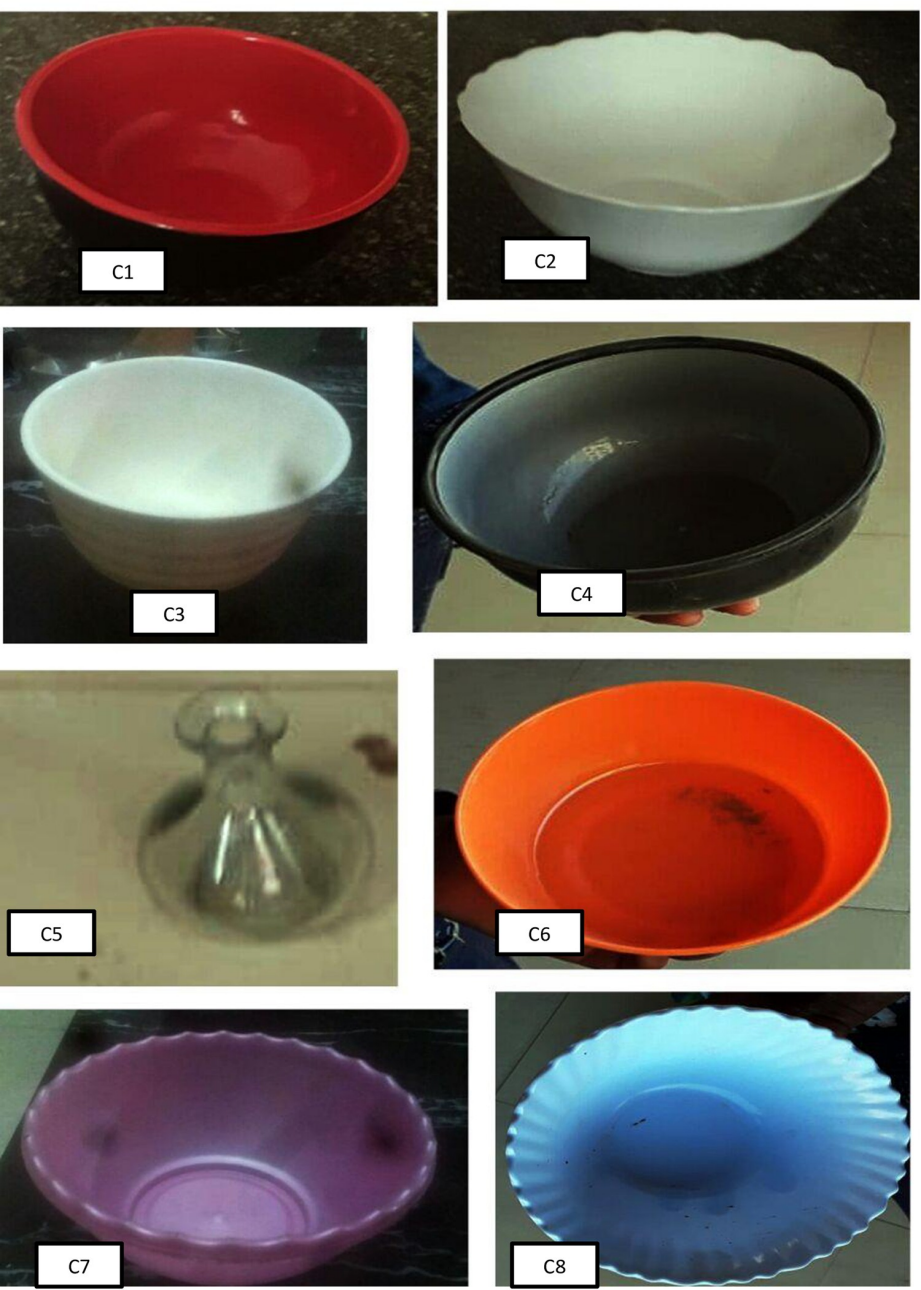


Initially, a survey was done among 21 lactating women and supermarkets, to identify the common food items and to take photographs of apparatuses that were typically used in the households. For each apparatus, a code was given for actual data collection. After coding the photographs of apparatuses, the actual data collection was started. The respondents were asked which apparatuses were used from the photo banner. Some food items like orange, mango, banana, and lemon were recorded in number, and size as large, medium, and small. For mixed dish foods, the respondents were asked to list all the food types and the ingredients (**Fig 1**).

### Data quality control

The data collection tool (Questionnaire) was prepared in English and translated into the local language (Amharic) and translated back into English to check its consistency. The data quality was maintained and assisted by a pretest, close supervision, and training of data collectors. In addition to this, the data quality was assured through checkups in data completeness at the field carefully every day, used photograph banners of household apparatuses for portion size estimation, and the digital food weighing scale was calibrated to zero during the standardization of the portion sizes of consumed food.

### Data processing and analysis

The data were entered and analyzed using EpiData version.3.0, and using IBM SPSS Statistics for Windows version 24.0 respectively. After the data were checked by Kolmogrove Smirnov and Shapiro Wilk test of normality, mean and standard deviation (mean, SD) were used to present normally distributed variables (p≥0.05), while the median and interquartile range was used to present skewed distribution (p<0.05). Both descriptive and binary logistic regression analysis was done.

The micronutrient value of each food item per 100 grams was obtained from the Ethiopian Food Composition Table (EFCT) [33, 34] and the World Food Composition Table [35, 36]. The World Food Composition Table (Kenya and Tanzania) was used to extract the micronutrient values of certain food items, and incomplete data on micronutrient values, which were not included in the Ethiopian Food Composition Table [35, 36]. In order to create a micronutrient database and to calculate the actual intake of the micronutrient; each food item that was consumed by respondents and the corresponding micronutrient values of the studied micronutrient must enter into the ESHA Food Processor Version 8.1 software. Finally, the calculated micronutrient values of each respondent by ESHA Food Processor software were transferred to excel and then to SPSS for analysis.

These studied micronutrients are selected based on their potential to decrease breast milk concentrations with low maternal intakes [37, 38], or the possible consequences arising from deficiencies for the health of both mothers and their infants [17, 39]. The estimated prevalence of inadequate intake of micronutrients (vitamin C, iron, niacin, riboflavin, thiamine, vitamin B_6_, vitamin B_12_ vitamin A, folate zinc, calcium selenium, and sodium) was calculated by NAR and the overall prevalence of nutrient intake was calculated by MAR. NAR of micronutrients and MAR of all micronutrients included in the study for lactating women were calculated by using the following formula [40]: This reflected what percentage of the recommended intake was consumed by lactating women in the last 24 hours. The RDA/AI of nutrients was taken from the Institute of Medicine the national academies press of America [41].


$$NAR=\frac{actual\;intake\;of\;micronutrient\;per\;day}{RDA\;of\;that\;micronutrient\;}\;$$ (Eq 1)

$$MAR=\frac{\sum NAR\;(each\;truncated\;at\;1)}{Number\;of\;micronutrients}$$ (Eq 2)

NAR was truncated at 1 so that a nutrient with a high NAR could not compensate for a nutrient with low NAR.

Both bivariable and multivariable binary logistic regression analyses were employed to identify the factors affecting overall micronutrient intake inadequacy among lactating women. Variables observed in bivariable binary logistic regressions (p≤0.25) were subsequently included in the multivariable binary logistic regression analysis to identify their independent effect. In multivariable binary logistic regression models, the Hosmer-Lemeshow goodness of fit test was performed for model fitness and a p-value >0.05 is a good fit. Variables with P-value less than 0.05 on multivariable binary logistic regression analysis were considered statistically significant factors. The strength of association between a dependent variable and independent variables was expressed by the Adjusted Odds Ratio (AOR). Then the final result was presented by texts, tables, and graphs.

### Ethical considerations

Ethical clearance (informed written consent) was obtained from the Ethical Review Board of Bahir Dar University, College of Medicine and Health Science, School of Public Health. Informed written permission was also obtained from the concerned authority of Bahir Dar city municipality administrative and the local government representative bodies of the selected kebeles. Oral consent was also secured from each lactating woman during data collection.

## Result

### Socio-demographic and socioeconomic characteristics of the respondents

Four hundred thirteen lactating women were involved in this study with a response rate of 92.3% and the median age of the respondents was 28±7.0 (median ±IQR). The highest level of education attained by respondents was college and above. One hundred fifty respondents (39.4%) were housewives, and 356(93.4%) were married. Concerning the food security status, 57(15%) of the respondents were food insecure. Regarding the wealth index of the respondents, 70(18.4%) and 117(30.7%) were the poorest and richest respectively (**Table 1**).

**Table 1:** Socio-demographic and socio-economic characteristics of lactating women in Bahir Dar city, Northwest Ethiopia, 2021(n = 381).

Variables and categories		Frequency	Percentage (%)
Age	From 20 to 30 years of age	250	65.6
	From 31 to 43 years of age	131	34.4
Educational status of lactating women	Cannot read and write	49	12.9
	primary school	85	22.3
	secondary school	88	23.1
	college and above	159	41.7
Husband educational status	Cannot read and write	19	5.2
	primary school	64	17.5
	secondary school	68	18.6
	college and above	214	58.6
The religion of lactating women	Orthodox	314	82.4
	Muslim	57	15.0
	Protestant	10	2.6
Occupational status of lactating women	Housewife	150	39.4
	Government employee	99	26.0
	Private employee	53	13.9
	Merchant	62	16.3
	Daily laborers and other	17	4.4
Occupational status of husband	Merchant	85	23.1
	Government employee	156	42.4
	Private employee	92	25
	Daily laborers and other	35	9.5
Marital status of lactating women	Married	356	93.4
	Divorced	23	6.0
	Widowed and other	2	0.6
Postpartum days/months	45 days to 6 month	103	27.1
	7 to 24 months	278	72.9
Number of children	<4	332	87.1
	≥4	49	12.9
Family size	<4	105	27.6
	≥4	276	72.4
Household food insecurity status	Food secure	324	85
	Food insecure	57	15
Wealth quintiles	Q1(poorest)	70	18.4
	Q2(poor)	85	22.3
	Q3(middle)	47	12.3
	Q4(rich)	62	16.3
	Q5(richest)	117	30.7

### Nutritional knowledge and health status of lactating women

Around 35% of respondents had poor nutritional knowledge scores regarding the requirement of additional meals for lactating women, frequency of extra meals, and the importance of iron-folic acid supplementation, source, and benefits of vitamins, and minerals. Concerning to illness of respondents, 24(6.3%) of lactating women were sick in the past months (**Table 2**).

**Table 2:** Nutritional knowledge and health status of lactating women in Bahir Dar city, Northwest Ethiopia, 2021(n = 381).

		Frequency	Percentage (%)
Nutritional knowledge of lactating women	Poor	133	34.9
	Medium	134	35.2
	Good	114	29.9
Visit health facility due to being sick/illness	No	357	93.7
	Yes	24	6.3

### Micronutrient intake of lactating women

Almost all micronutrient intakes except iron don’t meet the recommended level among lactating women. The median intake of vitamin A beta-carotene among respondents was 27.8 mcg. Similarly, the median intake of cobalamin (VB12), Vitamin C, calcium, iron, and folate was 0 mcg, 79.2 mg, 876.3 mg, 116.7 mg, and 645 mcg respectively. Approximately 98% of respondents had inadequate intake of vitamin A (failure to meet the RDA of vitamin A) (**Table 3**).

**Table 3:** Micronutrient intake of lactating women in Bahir Dar city, Northwest Ethiopia, 2021(n = 381).

Micronutrients	Median/IQR(Q1-Q3)	RDA/AI/EER	Prevalence of inadequacy (%) and (95% CI)
Vitamin A(mcg RAE)	27.8 ±(37.1)	1300	98.2(96.3, 99.3)
Vitamin B1(mg)	2.1 ±(0.9)	1.4	13.4(10.1, 17.2)
Vitamin B2(mg)	1.6 ±(0.7)	1.6	54.3(49.2, 59.4)
Vitamin B3(mg)	20.4 ±(15.7)	17	42.3(37.2, 47.4)
Vitamin B6(mg)	1.7 ±(1.1)	2	65.6(60.6, 70.4)
Vitamin B12(mcg)	0 ±(6.24)	2.8	68.5(63.6, 73.1)
Vitamin C(mg)	79.2 ±(88.9)	120	73.8(69, 78)
Folate (mcg)	645 ±(289.5)	500	25.2(20.9, 29.9)
Calcium (mg)	876.3 ±(318.7)	1000	70.9(66, 75.4)
Iron (mg)	110.2 ±(51.3)	10	0(0.00, 1.00)
Zinc (mg)	21.2 ±(11.4)	12	4.7(2.8, 7.4)
Selenium (mcg)	92.2 ±(81.6)	70	36(31.1,41.0)
Sodium (mg)	552.9 ±(582.4)	1500	96.1(88.4,94.2)
MAR(12 nutrients)			39.9(34.9, 45.0)

NB, MAR = mean adequacy ratio, mg = milligram, mcg = microgram, g = gram, CI = confidence interval

### Overall micronutrient intake inadequacy of lactating women

The prevalence of adequacy of iron was 100%; this implies that all respondents had NAR > 1, so it was excluded to calculate MAR. The overall prevalence of micronutrient intake inadequacy among lactating women was 39.9%. It was computed, from 12 micronutrients, after excluding iron.

### Factors affecting overall micronutrient intake inadequacy among lactating women

With multivariable binary logistic regression analysis; nutritional knowledge, marital status, and occupational status of lactating women were associated with overall micronutrient intake inadequacy. Being divorced was 2.7 times more likely to have overall micronutrient intake inadequacy than being married [AOR = 2.71, 95% CI (1.00, 7.33)]. The odds of overall micronutrient intake inadequacy were 2.6 higher in merchants than in housewives [AOR = 2.63, 95% CI (1.40, 4.93)]. Being a government employee was 2.5 times more likely to have overall micronutrient intake inadequacy than housewives [AOR = 2.45, 95% CI (1.37, 4.40)]. Lactating women who had poor nutritional knowledge were 2.7 times more likely to have overall micronutrient intake inadequacy than those who had good nutritional knowledge [AOR = 2.71, 95% CI (1.47, 4.99)] (**Table 4**).

**Table 4:** Bivariable and multivariable binary logistic regression analysis of overall micronutrient intake inadequacy among lactating women in Bahir Dar city, Northwest Ethiopia, 2021(n = 381).

		Overall Micronutrient Intake N (%)			
Variables	Category	Inadequate	Adequate	COR(95% CI)	AOR(95% CI)
Educational status of lactating women	Cannot read and write	26(17.1)	23(10.0)	1.87(.98, 3.56)*	.68(.247, 1.86)
	Primary school	34(22.4)	51(22.3)	1.10(.64, 1.89)	.91(.45, 1.85)
	Secondary school	32(21.1)	56(24.5)	.94(.55, 1.62)	.94(.49, 1.78)
	College and above	60(39.5)	99(43.2)	1	1
Educational status of husband (n = 365)	Cannot read and write	11(7.9)	8(3.6)	2.30(.89, 5.97)*	1.42(.34, 5.63)
	Primary school	24(17.1)	40(17.8)	1.05(.56, 1.79)	.92(.40, 2.16)
	Secondary school	25(17.9)	43(19.1)	.97(.55, 1.71)	.92(.47, 1.83)
	College and above	80(57.1)	134(59.6)	1	1
Occupational status of lactating women	Housewife	47(30.9)	103(45.0)	1	1
	Government employee	44(28.9)	55(24.0)	1.75(1.04, 2.97)**	2.45(1.37, 4.40)**
	Private employee	16(10.5)	37(16.2)	.95(.48, 1.87)	.76(.37, 1.55)
	Merchant	35(23.0)	27(11.8)	2.84(1.55, 5.22)**	2.63(1.40, 4.93)**
	Daily laborers and other	10(6.6)	7(3.1)	3.13(1.12, 8.73)	1.66(.54, 5.10)
Marital status of lactating women	Married	136(89.5)	222(96.9)	1	1
	Divorced	16(10.5)	7(3.1)	3.73(1.50, 9.30)**	2.71(1.01, 7.33)**
Knowledge status of lactating women	Poor knowledge	68(44.7)	65(28.4)	2.18(1.30, 3.66)**	2.71(1.47, 4.99)**
	Medium knowledge	47(30.9)	87(38.0)	1.12(.66, 1.91)	1.25(.72, 2.19)
	Good knowledge	37(24.3)	77(33.6)	1	1
HFIAS	Food secure	31(20.4)	26(11.4)	1	1
	Food insecure	121(79.6)	203(88.6)	2.00(1.13, 3.53)**	.83(.34, 2.03)
Wealth Index	Poorest	37(24.3)	33(14.4)	1.73(.95, 3.15)*	.86(.40, 1.84)
	Poor	28(18.4)	57(24.9)	.76(.42, 1.36)	.76(.41, 1.42)
	Middle	22(14.5)	25(10.9)	1.36(.69, 2.69)	1.39(.68, 2.84)
	Rich	19(12.5)	43(18.8)	.68(.35, 1.31)	.68(.35, 1.34)
	Richest	46(30.3)	71(31.0)	1	1

Note

* significant at p 0.25

** Significant at p< 0.05. Unmarked not significant, 1 = reference group, COR = crude odds ratio, AOR = adjusted odds ratio, CI = confidence interval

## Discussion and conclusion

The overall prevalence of micronutrient intake inadequacy among lactating women was 39.9% in the present study area. A similar report revealed that the mean inadequacy ratio was 36% among 0 to 6 months postpartum lactating women in Nairobi [42]. The overall mean population prevalence of micronutrient inadequacy was 43% in Indonesia among 2 to 5 months postpartum lactating women [5]. This higher inadequacy intake was due to the main food sources of lactating women being starchy staples can place which lead them to risk of micronutrient deficiencies and monotonous foods that lack diversity. Including foods in the diet, which have high micronutrient density such as pulses or legumes, vegetables (including green leafy vegetables), and fruits are the preferred way of ensuring optimal nutrition including micronutrient adequacy for most population groups. Most population groups afflicted by micronutrient deficiency largely subsist on refined cereal grain or tuber-based diets, which provide energy and protein, but are insufficient in critical micronutrients.

Insufficient intake of vitamin A among lactating women was 98.2%. A similar finding has been reported among lactating women in Zambia 99.9% [11], although even lower estimates have been reported in earlier studies such as Niger 88.8% [10], Iran 51.4% [43], and China 94.5% [8]. The difference may be due to the seasonal variability and low consumption of vitamin A food sources such as green leafy vegetables in the present study area.

This study found that the intake of several important vitamins among lactating women was 13%~74% lower than recommended, including thiamine, riboflavin, niacin, pyridoxine, cobalamin, and vitamin C. Similar findings are also reported in Niger [10] (usual dietary intakes of thiamin, riboflavin, niacin, vitamin B12, and vitamin C were inadequate among >50% of lactating women), Zambia [11] (the inadequate intake of thiamin, riboflavin, niacin, vitamin B12, and vitamin C ranges from 22 to 77.6% among lactating women), and Indonesia [5] (the mean prevalence of adequacy among lactating women was less than 60% for six micronutrients, most notably for vitamins B6, and C, followed by niacin, calcium, B12, and vitamin A). This was linked to the low intakes of fresh fruits and vegetables, the consumption of meat/flesh foods was also relatively low and contributed to the low adequacy of intakes of vitamins B6, B12, and niacin by these lactating women, the difference may be due to the diets of the lactating women mainly on cereals and grains, which provide high protein and carbohydrate, but lacks essential micronutrient. Likewise, inadequate intake of folate was 25.2% among lactating women. The result of this study was comparable in Indonesia (21%) [5]. On the other hand, it was lower than in different reports such as Zambia (74.6%) [11], Niger (83%) [10], Iran (61.4%) [43], and Brazilian (72%) [14]. This may be due to the high consumption of plant-based foods in this study area especially cereals and legumes.

Inadequate calcium intake (below recommended) was evident in lactating women (70.9%). A consistent finding was reported in Nigeria (66.1%) [9]. However, the report of other findings was lower compared to this result in Iran (38.6%) [43]. The inadequate intake of our findings was lower than the reports of Zambia 99.9% [11], Niger 100% [10], Brazil 92% [14], Indonesia 81% [6], and China 84.8% [8]. This may be due to the low consumption of dairy products including milk which was the major source of calcium in the present study area.

Surprisingly, the iron intake of lactating women was high. Sufficient intake of iron also has been reported in different studies [9, 10, 23, 43]. But inadequate intake was reported in Zambia (37.1%) [11], Brazil (15.7%) [14], and Shangi of China (48.3%) [13]. This could be so because the iron requirements during lactation decreased from 27mg/day in pregnancy to merely 9mg/day, compared to pre-pregnancy amounts of 18mg/day and breastfeeding usually suppresses menstruation for a few months, thus minimizing iron losses [44]. This difference may be due to the high consumption of cereals and grains and legumes in the study area which is the major source of iron, and it did not consider the bioavailability of iron.

Approximately 95% of lactating women had adequate intake of zinc. This study was supported by similar findings [5, 6]. But higher inadequate intake of zinc among lactation was reported [10, 11, 13, 14, 43]. This implied that the intake observed could only be apparent since bioavailability was not taken into consideration and the intake of these nutrients was mainly from plant sources which have been shown to contain low bioavailability in this study area.

Overall, the dietary intake in lactating women was lower than recommended. With the exception of iron, the intakes of micronutrients among the respondents were lower than recommended. Therefore; micronutrient deficiencies are public health concerns in Bahir Dar City, partly due to monotonous, cereal and legume-based diets. With multivariable binary logistic regression analysis, Nutritional knowledge, marital status, and occupational status were the significant factors that have been associated with the overall micronutrient intake inadequacy of lactating women.

## Abbreviations

AI: Adequate Intake
FAO: Food and Agriculture Organization of the United Nations
HFIAS: Household Food Insecurity Accesses Scale
IQR: Interquartile Range
MAR: Mean Adequacy Ratio
NAR: Nutrient Adequacy Ratio
RDA: Recommended Dietary Allowance
RNI: Reference Nutrient Intake
